# Real-World Experiences with the Combination Treatment of Ledipasvir plus Sofosbuvir for 12 Weeks in HCV Genotype 1-Infected Japanese Patients: Achievement of a Sustained Virological Response in Previous Users of Peginterferon plus Ribavirin with HCV NS3/4A Inhibitors

**DOI:** 10.3390/ijms18050906

**Published:** 2017-04-25

**Authors:** Tatsuo Kanda, Shin Yasui, Masato Nakamura, Eiichiro Suzuki, Makoto Arai, Yoshihiko Ooka, Sadahisa Ogasawara, Tetsuhiro Chiba, Tomoko Saito, Yuki Haga, Koji Takahashi, Reina Sasaki, Shuang Wu, Shingo Nakamoto, Akinobu Tawada, Hitoshi Maruyama, Fumio Imazeki, Naoya Kato, Osamu Yokosuka

**Affiliations:** 1 Department of Gastroenterology, Graduate School of Medicine, Chiba University, 1-8-1 Inohana, Chuo-ku, Chiba 260-8670, Japan; ntcph863@yahoo.co.jp (S.Y.); nkmr.chiba@gmail.com (M.N.); eiichiro0709@hotmail.com (E.S.); araim-cib@umin.ac.jp (M.A.); ooka-y@umin.ac.jp (Y.O.); sadahisa@me.com (S.O.); techiba@faculty.chiba-u.jp (T.C.); gcmmg242@yahoo.co.jp (T.S.); hagayuki@gmail.com (Y.H.); koji517@gmail.com (K.T.); reina_sasaki_0925@yahoo.co.jp (R.S.); gosyou100@yahoo.co.jp (S.W.); maru-cib@umin.ac.jp (H.M.); kato-2im@ims.u-tokyo.ac.jp (N.K.); yokosukao@faculty.chiba-u.jp (O.Y.); 2 Department of Molecular Virology, Graduate School of Medicine, Chiba University, 1-8-1 Inohana, Chuo-ku, Chiba 260-8670, Japan; nakamotoer@yahoo.co.jp; 3 Safety and Health Organization, Chiba University, 1-33 Yayoicho, Inage-ku, Chiba 263-8522, Japan; akinobutawada@hospital.chiba-u.jp (A.T.); imazekif@faculty.chiba-u.jp (F.I.)

**Keywords:** genotype 1, hepatitis C virus, ledipasvir, NS3/4A inhibitors, sofosbuvir

## Abstract

The aim of this study was to characterize the treatment response and serious adverse events of ledipasvir plus sofosbuvir therapies in Japanese patients infected with hepatitis C virus (HCV) genotype 1 (GT1). This retrospective study analyzed 240 Japanese HCV GT1 patients treated for 12 weeks with 90 mg of ledipasvir plus 400 mg of sofosbuvir daily. Sustained virological response at 12 weeks post-treatment (SVR12) was achieved in 236 of 240 (98.3%) patients. Among treatment-naïve patients, SVR12 was achieved in 136 of 138 (98.6%) patients, and among treatment-experienced patients, SVR12 was achieved in 100 of 102 (98.0%) patients. In patients previously treated with peginterferon plus ribavirin with various HCV NS3/4A inhibitors, 100% SVR rates (25/25) were achieved. Two relapsers had HCV NS5A resistance-associated variants (RAVs), but no HCV NS5B-S282 was observed after they relapsed. We experienced two patients with cardiac events during treatment. In conclusion, combination of ledipasvir plus sofosbuvir for 12 weeks is a potential therapy for HCV GT1 patients. Caution is needed for HCV NS5A RAVs, which were selected by HCV NS5A inhibitors and cardiac adverse events.

## 1. Introduction

Hepatitis C virus (HCV) infection constitutes great threats to public health globally, and leads to a large number of deaths every year. Hepatocellular carcinoma (HCC) has a poor prognosis, and the 5- and 10-year survival rates of HCC in Japan are 34% and 16%, respectively [1]. HCV causes ~70% of HCC in Japan [2]. It is important to eradicate HCV at the early stage of infection as well as to diagnose HCC early [3,4]. Eradication of HCV by interferon prevents the occurrence of HCC and development of liver fibrosis, providing survival benefits for patients infected with HCV [5,6]. Therefore, it is important for patients with HCV infection to eradicate this virus.

Interferon-free treatment with the combination of direct-acting antivirals (DAAs) against HCV has a higher efficacy and fewer adverse events than interferon-including treatment [7]. In Japan, HCV genotype 1b (GT1b) occupied 70% of HCV infection and HCV GT1a is rare [8]. The combination of the HCV NS3/4A inhibitor asunaprevir and the HCV NS5A inhibitor daclatasvir for 24 weeks was the first approved interferon-free treatment against HCV GT1b patients in July, 2014 [7,9]. Although several guidelines recommended the measurement of HCV NS5A resistance-associated variants (RAVs) before this treatment, the treatment failure of the combination of asunaprevir and daclatasvir introduced multiple RAVs into the HCV NS3/4A and NS5A regions [10,11]. We do not use the combination of asunaprevir and daclatasvir for HCV GT1 patients with peginterferon plus ribavirin with NS3/4A protease simeprevir failure [12], because 66.7% of patients with prior peginterferon plus ribavirin with simeprevir failure had virological failure in the retreatment with the combination of asunaprevir and daclatasvir [9].

The combination of HCV NS5A inhibitor ledipasvir and HCV NS5B inhibitor sofosbuvir with or without ribavirin is a highly effective regimen in HCV GT1 patients with or without cirrhosis [13,14,15,16,17]. Combination use of ledipasvir and sofosbuvir for the treatment of HCV GT1 also showed 91–100% sustained virological response (SVR) in HCV GT1 patients who are treatment-naïve [15] and treatment-experienced [14], with or without ribavirin.

In Japan, the combination of ledipasvir and sofosbuvir without ribavirin for 12 weeks was the second approved interferon-free regimen for HCV GT1 patients in August, 2015 [6]. In the present study, we retrospectively examined the effect of the combination treatment of ledipasvir and sofosbuvir for 12 weeks in real-world patients infected with HCV GT1 in Japan and showed that the combination of ledipasvir and sofosbuvir is highly effective for HCV GT1 patients. We also demonstrated that the combination of ledipasvir and sofosbuvir is effective for HCV GT1 Japanese patients with treatment failure of peginterferon plus ribavirin with HCV NS3/4A protease.

## 2. Patients and Methods

### 2.1. Patients

A total of 240 consecutive HCV GT1-infected patients who commenced 12-week treatments with ledipasvir (90 mg) and sofosbuvir (400 mg) daily (fixed-dose compound: Havoni, Gilead Sciences, Tokyo, Japan) and in whom SVR12 was judged between September 2015 and March 2017 at Chiba University Hospital were included (Table 1).

A total of 138 treatment-naïve patients and 102 interferon-treated patients were included. Eligible patients were 20 years of age and older and were infected with HCV GT1 at the baseline. The exclusion criteria were as follows: (1) Child-Pugh B or C cirrhosis; (2) severe anemia at baseline; (3) severe renal dysfunction at baseline; (4) presence of HCC; and (5) any serious medical condition of any other organ, such as arrhythmia or congestive heart failure. Patients with a history of curative treatment of HCC were included.

This retrospective study was approved by the Ethics Committee of Chiba University, School of Medicine (numbers 1462 and 1753). Participation in the study was posted at our institutions. Informed consent was obtained from all patients, and this study conformed to the ethical guidelines of the Declaration of Helsinki.

### 2.2. Clinical and Laboratory Assessments

Clinical parameters were measured by standard laboratory techniques at a central laboratory in Chiba University Hospital. Blood samples were obtained at the baseline and weeks 4, 8 and 12 and then 4, 8 and 12 weeks after the end of treatment. HCV serotyping and genotyping were performed as previously described [12]. HCV RNA was measured by COBAS TaqMan HCV assay version 2.0 (Roch Diagnostics, Tokyo, Japan) with a lower limit of quantification of 15 IU/mL (=1.2 LIU/mL). Rapid virological response (RVR) was defined as undetectable HCV RNA at week 4 after the start of treatment. End-of-treatment response (EOTR) was defined as undetectable HCV RNA at the end of treatment. SVR at 4, 8, or 12 weeks (SVR4, SVR8, or SVR12, respectively) was also used to evaluate the virological response.

HCV NS5A resistance-associated variants (RAVs) at L31 and Y93 and HCV NS5B RAV at S282 were determined by a commercial direct-sequencing assay (SRL Laboratory, Tokyo, Japan) [12].

Cirrhosis was diagnosed by a previous liver biopsy, transient elastography (Fibroscan of greater than 12 kPa) and/or ultrasonography (sign of cirrhosis). HCC was excluded by imaging modalities such as ultrasonography, computed tomography (CT), and/or gadolinium ethoxybenzyl diethlenetriamine pentaacetic acid (Gd-EOB-DTPA) enhanced magnetic resonance imaging (MRI).

### 2.3. DNA Extraction and IL28B Genotyping

In some patients, we analyzed IL28B rs8099917 as the major genotype and TG/GG as the minor genotytype as previously described [18]. To extract a DNA sample from blood cells, we used DNA Extract All Lysis Reagents (Applied Biosystems Inc., Foster City, CA, USA). A specific TaqMan genotyping assay was performed for IL28B rs8099917 [18]. This protocol was approved by the Ethics Committee of Chiba University, School of Medicine (number 582).

### 2.4. Statistical Analysis

Data are expressed as the mean ± standard deviation (SD). Statistical analyses were performed by univariate analyses using Student’s *t*-test or a Chi-squared test. *p* < 0.05 was considered statistically significant. Statistical analysis was performed using Excel Statistics program for Windows 2010 (SSRI, Tokyo, Japan).

## 3. Results

### 3.1. Patient Characteristics

Demographic and baseline characteristics by previous treatment status are shown in Table 1. The mean age was 65.8 years and 145 (60.4%) participants were ≥65 years old. Six, 206 and 28 were positive for HCV GT1a, GT1b and GT1, respectively. Forty-three (14.2%) underwent curative treatment for HCC, and 87 (36.3%) had cirrhosis. Of the 240 patients examined, 138 (57.5%) were treatment-naïve and 102 (42.5%) were interferon treatment-experienced. Of these 102 patients, 26 patients had experienced DAA-including regimens; 25 received peginterferon plus ribavirin with HCV NS3/4A inhibitors (16, simeprevir; 4, telaprevir; 3, faldaprevir; and 2 vaniprevir); and one received HCV NS3/4A inhibitor asunaprevir plus HCV NS5A inhibitor daclatasvir for 2 weeks before discontinuing [12]. In 76 interferon-treatment-experienced patients who were not previously treated by DAAs, the previous treatment responses were as follows: 29, null response; 25, relapse; 14, discontinuation due to adverse events; 2, viral breakthrough; and 6, unknown.

### 3.2. Treatment Response and Efficacy of Combination Treatment with Ledipasvir plus Sofosbuvir

Only one patient discontinued the fixed-dose compound at 3 days due to his arrhythmia. Another 239 (99.6%) patients continued the combination treatment of ledipasvir plus sofosbuvir for 12 weeks, and adherence to these drugs was much better than that for the combination treatment of HCV NS3 inhibitor asunaprevir plus HCV NS5A inhibitor daclatasvir for 24 weeks as we previously reported [12]. The rapid virological response (RVR) and end-of-treatment response (EOTR) rates were 73.8% (177/240) and 99.6% (239/240), respectively (Table 2). The rates of SVR4, SVR8 and SVR12 were 99.2% (238/240), 98.3% (236/240) and 98.3% (236/240), respectively.

**Figure 1:**
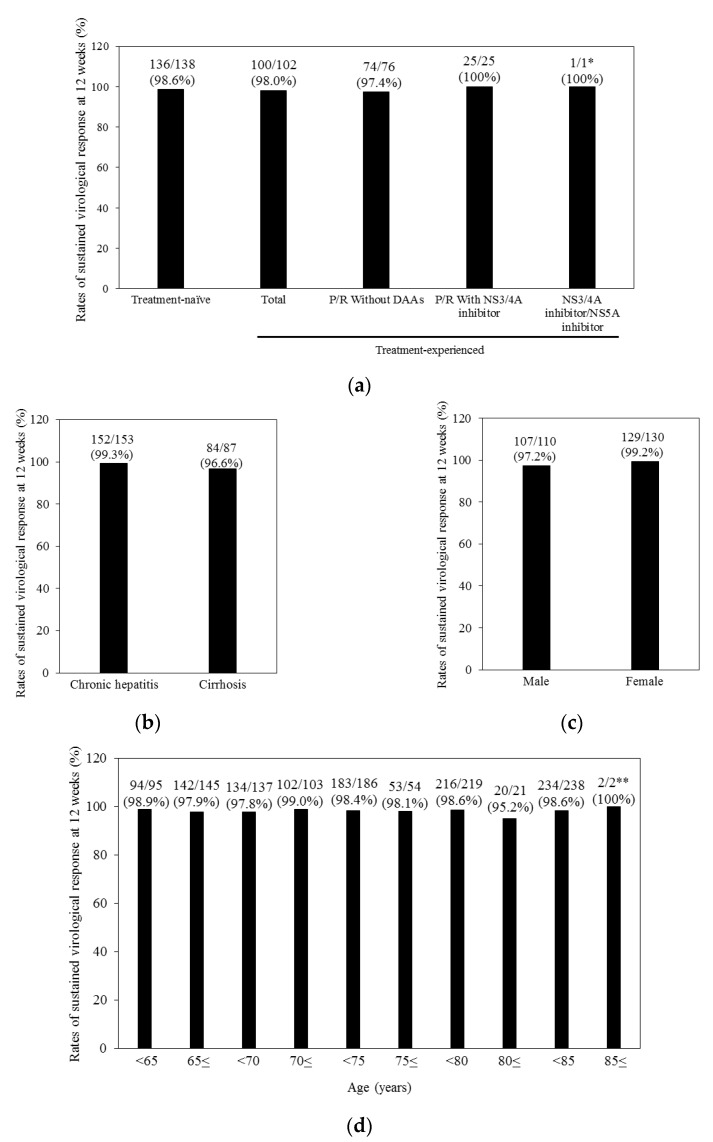

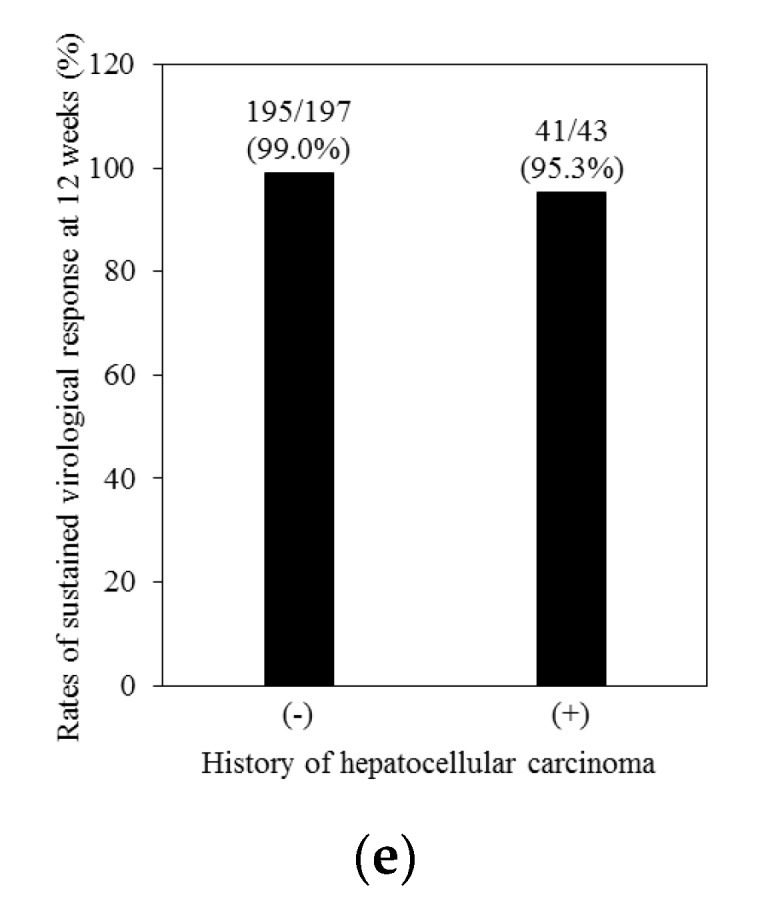
Sustained virological response at 12 weeks (SVR12) rates for the various groups. (**a**) Previous interferon treatment; (**b**) Chronic hepatitis or cirrhosis; (**c**) Gender; (**d**) Age; and (**e**) History of hepatocellular carcinoma. P, peginterferon; R, ribavirin; and DAAs, direct-acting antivirals against HCV. * *p* < 0.01 vs. other groups; ** *p* < 0.01 vs. age <85 group.

Figure 1 shows the SVR12 rates according to the various categories. The SVR rates of treatment-naïve (98.6%) participants were not statistically significantly different from those of treatment-experienced patients (98.0%) (Figure 1a). Of interest, 100% of patients with previous treatment failure of peginterferon plus ribavirin with various HCV NS3/4A inhibitors achieved SVR. In the present study, we treated only one patient who discontinued the combination of asunaprevir plus daclatasvir at 2 weeks due to cough [12], and he also achieved SVR.

Unlike the previous standard of care consisting of peginterferon plus ribavirin therapies [19], the combination treatment of ledipasvir plus sofosbuvir for 12 weeks could lead to high SVR rates in cirrhotic patients, compared with non-cirrhotic patients (statistically not significant (N.S.)) (Figure 1b). We did not find any differences in the SVR rates between different genders (Figure 1c). Elderly patients aged equal to and more than 85 years could also achieve significantly higher SVR12 (*p* < 0.01) (Figure 1d). If curative treatment for HCC was performed, a history of HCC did not affect their SVR12 (N.S.) (Figure 1e).

### 3.3. Analysis of Resistance-Associated Variants (RAVs) in Relapsers to Ledipasvir plus Sofosbuvir

We analyzed HCV NS5A and NS5B RAVs after treatment failure in two treatment relapsers (Table 3). We detected these RAVs by commercial direct sequence assays. The patient with relapse at 4 weeks post-treatment had two HCV NS5A L31 and Y93 mutants. The patient with relapse at 8 weeks post-treatment only had one HCV NS5A L31 mutant. These two patients did not have NS5B-S282. Of interest, these two patients were interferon-null responders and had cirrhosis, and one had a history of curative treatment for HCC. Unfortunately, the IL28B rs8099917 genotype was not determined in patient no. 2. However, patient no. 1 had the IL28B rs8099917 TT genotype (major genotype).

### 3.4. Safety in Ledipasvir plus Sofosbuvir Treatment

Serious adverse events were observed in three patients. One patient discontinued the fixed-dose compound at 3 days due to ventricular tachycardia. Another patient had angina after 11 weeks of the treatment with ledipasvir plus sofosbuvir. By coronary angiography with the acetylcholine provocation test, she was diagnosed with vasospastic angina, but she continued antiviral treatment and achieved SVR12. After ~1 month after end of treatment (EOT), one patient died due to a subarachnoid hemorrhage, which appeared one day after end of treatment.

## 4. Discussion

We examined the effect of the combination treatment of ledipasvir and sofosbuvir for 12 weeks in HCV GT1 patients in Japan, and confirmed the effectiveness and safety of this DAA combination for the treatment of HCV GT1 not only in naïve patients, but also previously treated individuals. Of note, 100% SVR12 has been achieved in patients who did not have SVR with peginterferon plus ribavirin with various HCV NS3/4A inhibitor treatments. In addition, HCV NS5A RAVs and cardiac adverse events were observed during the treatment.

In our previous study, we demonstrated naturally occurring RAVs of HCV NS5A inhibitors by ultra-deep sequencing and that HCV NS5A Y93H are found in ~30% of HCV NS5A inhibitor-treatment-naïve patients with the chronic HCV genotype 1b in our area [20]. Although the present study included one patient who was previously treated with a HCV NS3/4A inhibitor plus HCV NS5A inhibitor for only 2 weeks, the combination treatment of ledipasvir plus sofosbuvir for 12 weeks on HCV genotype 1-infected Japanese patients was highly effective. It has been reported that the presence of baseline NS5A RAVs does not impact the treatment outcome in HCV GT1b patients treated with ledipasvir plus sofosbuvir for 12 weeks if they were not previously treated with HCV NS5A inhibitors [21]. Pretreatment ledipasvir-specific RAVs impact the treatment outcome in some patient groups such as treatment-experienced patients with HCV GT1a [22].

We observed two cirrhotic relapsers with sofosbuvir plus ledipasvir treatment and who had HCV NS5A RAVs after relapse. Although the pre-existing HCV NS5A and NS5B RAVs data were not available in the present study, HCV NS5A RAVs may undermine the effectiveness of sofosbuvir plus ledipasvir in cirrhotic patients with HCV GT1b [23]. This might be the reason for the different SVR rates between cirrhotic and non-cirrhotic patients (Figure 1b), since a previous study [23] showed that the NS5A RAVs lead to lower SVR12 in patient with cirrhosis. Recent reports demonstrated that the SVR rate was only 69% when treated with ledipasvir plus sofosbuvir therapy in patients with prior asunaprevir plus daclatasvir treatment [24]. Prior asunaprevir plus daclatasvir treatment is associated with failure of ledipasvir plus sofosbuvir therapy due to multiple HCV NS5A RAVs.

Akuta et al. [25] also reported that the SVR rate was only 65% for those treated by ledipasvir plus sofosbuvir therapy in 43 patients with prior asunaprevir plus daclatasvir treatment (11 nonresponders, 16 viral breakthroughs, and 16 relapsers). They also reported that ledipasvir plus sofosbuvir therapy resulted in 100% SVR rates in seven patients who discontinued asunaprevir plus daclatasvir treatment due to adverse events, which our results supported. Deferral of treatment should be recommended, pending the availability of new drugs for patients with HCV GT1 in whom previous treatment with any HCV NS5A inhibitors failed, in those who have cirrhosis and HCV NS5A RAVs or do not have cirrhosis [10,26,27], although sofosbuvir plus ledipasvir may retain partial activity on HCV NS5A RAVs that were selected by HCV NS5A inhibitors [28,29].

RVR rates in the present study were 73.8%, lower than the RVR rates in the previous studies [13,14,15,17,30,31]. This may be the result of a more sensitive detection method of the present study. According to the European Association for the Study of the Liver (EASL) guidelines in 2011 [32], the RVR definition for HCV consists of HCV RNA levels <50 IU/mL. As the techniques used for HCV RNA detection have recently improved, we used the definition of RVR consists of HCV RNA levels <15 IU/mL according to the new EASL guidelines in 2014 [33].

In treatments with DAAs, targets of DAAs affect the treatment response. In the present study, 100% of patients with previous treatment of peginterferon plus ribavirin with a HCV NS3/4A inhibitor achieved SVR with the combination treatment of ledipasvir plus sofosbuvir. These combination targets of HCV NS5A and NS5B without NS3/4A have a potential effectiveness for patients with previous treatment failure of peginterferon plus ribavirin with a HCV NS3/4A inhibitor, such as simeprevir.

We experienced one patient with ventricular tachycardia and discontinued the treatment combination at 3 days after the commencement of treatment, and one other patient had vasospastic angina. Neither patient took amiodarone [34,35,36]. The pathophysiological mechanism underlying the cardiac adverse event in our patients is not clear. However, the potential cardiac toxicity of sofosbuvir-containing regimens suggests the need for caution with the combination regimens of ledipasvir plus sofosbuvir as well as careful monitoring of cardiac rhythm during the initiation of therapy.

## 5. Conclusions

Combination treatment of ledipasvir plus sofosbuvir for 12 weeks is highly effective for treatment-naïve patients and patients who were previously treated with peginterferon plus ribavirin with or without a HCV NS3/4A inhibitor and who were infected with HCV GT1. Caution is needed for HCV NS5A RAVs that were selected by HCV NS5A inhibitors and cardiac adverse events.

## Figures and Tables

**Table 1:** Characteristics of 240 hepatitis C virus (HCV) genotype (GT)-1 patients at the start of treatment.

Characteristics	All (*n* = 240)	Treatment-Naïve (*n* = 138)	Treatment-Experienced (*n* = 102)	*p*-Values ^1^
Age (years)	65.8 ± 11.6	67.5 ± 11.3	63.5 ± 11.6	0.00787
Gender (male/female)	110/130	59/79	51/51	0.328
Interferon (naive/experienced)	138/102	138/0	0/102	N.A.
HCV GT (1a/1b/1)	6/206/28	4/120/14	2/86/14	0.994
HCV RNA (L/H)	22/218	20/118	2/100	0.00194
Body weight (kg)	56.9 ± 10.2	55.9 ± 11.1	58.2 ± 8.8	0.0851
Body length (cm)	160 ± 9.5	160 ± 9.3	161 ± 9.7	0.420
History of HCC +/−	43/197	26/112	17/85	0.792
Chronic hepatitis/cirrhosis	153/87	88/50	65/37	0.897
Liver stiffness (kPa)	11.4 ± 12.7	10.8 ± 12.4	12.2 ± 13.1	0.399
AST (IU/L)	51.4 ± 32.0	48.1 ± 24.2	55.9 ± 39.9	0.0617
ALT (IU/L)	46.9 ± 40.2	41.6 ± 26.9	54.0 ± 52.5	0.0179
Hemoglobin (g/dL)	13.4 ± 1.6	13.4 ± 1.5	13.5 ± 1.6	0.620
Platelets (×10^4^/μL)	15.3 ± 6.8	15.7 ± 7.5	14.9 ± 5.7	0.368
eGFR (mL/min/1.73 m^2^)	74.4 ± 17.9	73.2 ± 16.7	76.3 ± 19.6	0.188

Data are expressed as the mean ± standard deviation (SD). HCC, hepatocellular carcinoma; HCV RNA: L, <5.0 LIU/mL and H, ≥5.0 LIU/mL; AST, aspartate aminotransferase; ALT, alanine aminotransferase; eGFR, estimated glomerular filtration rates; N.A., not available. ^1^ *p*-values, treatment-naïve versus treatment-experienced groups.

**Table 2:** Response during and after treatment.

Characteristics	All (*n* = 240)	Treatment-Naïve (*n* = 138)	Treatment-Experienced (*n* = 102)	*p*-Values ^1^
HCV undetectable no. (%)				
*During treatment*				
At 4 w	177 (73.8)	106 (76.8)	71 (69.6)	0.221
At 8 w	237 (98.8)	136 (98.6)	101 (99.0)	0.791
At 12 w	239 (99.6)	137 (99.3)	102 (100)	0.879
*After treatment*				
Post 4 w	238 (99.2)	137 (99.3)	101 (99.0)	0.615
Post 8 w	236 (98.3)	136 (98.6)	100 (98.0)	0.838
Post 12 w	236 (98.3)	136 (98.6)	100 (98.0)	0.838
Virological failure				
Discontinuation	1	1/138 (0.7)	0/102 (0)	0.879
Relapse	2	0/138 (0)	2/102 (2.0)	0.350
Lost due to AEs	1	1/138 (0.7)	0/102 (0)	0.879

AEs, adverse events; w, weeks. ^1^ *p*-values, treatment-naïve versus treatment-experienced groups.

**Table 3:** Two patients who failed to respond to sofosbuvir plus ledipasvir treatment.

No.	Age/Gender	Previous Treatment Response	GT	Cirrhosis/HCC	Efficacies	Adherence >80%	NS5A-L31	NS5A-Y93	NS5B-S282
1	66/Male	PegIFN/RBV null response	1b	Yes/+	Relapse (post 4 w)	Yes	M	M	W
2	58/Male	IFN null response	1b	Yes/−	Relapse (post 8 w)	Yes	M	W	W

PegIFN/RBV, peginterferon plus ribavirin; GT, genotype; HCC, previous curative treatment of hepatocellular carcinoma; M, mutation; and W, wild-type. Resistance-associated variants (NS5A-L31 and Y93 and NS5B-S282) after treatment-relapse were determined by direct-sequence methods. Patient no. 2 took a proton pump inhibitor during treatment.
